# Monitoring Blood Immune Cells in Patients with Advanced Small Cell Lung Cancer Undergoing a Combined Immune Checkpoint Inhibitor/Chemotherapy

**DOI:** 10.3390/biom13020190

**Published:** 2023-01-17

**Authors:** Dagmar Riemann, Steffi Turzer, Georgi Ganchev, Wolfgang Schütte, Barbara Seliger, Miriam Möller

**Affiliations:** 1 Institute of Medical Immunology, Martin Luther University Halle-Wittenberg, 06112 Halle, Germany; 2 Clinic of Internal Medicine, Hospital Martha-Maria Halle-Dölau, 06120 Halle, Germany

**Keywords:** biomarker, dendritic cells, HLA-DR^low^ monocytes, immune checkpoint inhibitor, immune monitoring, neutrophil/lymphocyte ratio, overall survival, slan+ non-classical monocytes, small-cell lung cancer

## Abstract

In this exploratory prospective observational study on 40 small cell lung cancer (SCLC) patients treated with a combination of chemotherapy and immune checkpoint inhibitors, blood immune cells were characterized by multi-color flow cytometry at the baseline and at the third therapy cycle. The numbers of neutrophils and of T-, B-, and NK cells, as well as the frequency of HLA-DR^low^ monocytes, 6-SulfoLacNAc (slan)+ non-classical monocytes and circulating dendritic cell (DC) subtypes were determined. The prognostic value of the parameters was evaluated by the patient’s survival analysis with overall survival (OS) as the primary endpoint. In addition, blood cell parameters from SCLC patients were compared to those from non-SCLC (NSCLC). The global median OS of patients was 10.4 ± 1.1 months. Disease progression (15% of patients) correlated with a higher baseline neutrophil/lymphocyte ratio (NLR), more HLA-DR^low^ monocytes, and lower NK cell and DC numbers. The risk factors for poor OS were the presence of brain/liver metastases, a baseline NLR ≥ 6.1, HLA-DR^low^ monocytes ≥ 21% of monocytes, slan+ non-classical monocytes < 0.12%, and/or CD1c+ myeloid DC < 0.05% of leukocytes. Lymphocytic subpopulations did not correlate with OS. When comparing biomarkers in SCLC versus NSCLC, SCLC had a higher frequency of brain/liver metastases, a higher NLR, the lowest DC frequencies, and lower NK cell numbers. Brain/liver metastases had a substantial impact on the survival of SCLC patients. At the baseline, 45% of SCLC patients, but only 24% of NSCLC patients, had between three and five risk factors. A high basal NLR, a high frequency of HLA-DR^low^ monocytes, and low levels of slan+ non-classical monocytes were associated with poor survival in all lung cancer histotypes. Thus, the blood immune cell signature might contribute to a better prediction of SCLC patient outcomes and may uncover the pathophysiological peculiarities of this tumor entity.

## 1. Introduction

Small-cell lung cancer (SCLC) is an aggressive neuroendocrine carcinoma that constitutes about 13–15% of all lung cancers [1]; two-thirds of cases occur in an advanced stage. Despite chemotherapy sensitivity, patients often rapidly progress, and overall survival (OS) is poor. In recent years, the addition of immune checkpoint inhibitor (ICI) therapy to frontline platinum-based chemotherapy has modestly improved the median survival of patients with extended-stage SCLC, and this combination is approved as a standard of care [2,3,4,5]. 

The identification of the baseline characteristics of patients, who will most benefit from treatment with chemo/immunotherapy, remains an important challenge. The biomarker-driven categorization of therapy responders and non-responders would minimize unnecessary exposure of patients to potentially permanent immune-related toxicities and reduce the financial burden of health systems due to these expensive treatments [6]. In non-SCLC (NSCLC), the suggested biomarkers for ICI therapy are PD-L1 expression of tumor tissue, tumor mutational burden, and DNA mismatch repair deficiency/microsatellite instability (for review, see [7]). PD-L1 expression is less prevalent in SCLC than in NSCLC [8]. In randomized studies, PD-L1 has not been shown to be predictive of the response to ICI therapy [9,10]. In addition, the CheckMate 331 trial demonstrated that the tumor mutational burden did not predict clinical outcomes [10]. On the other hand, the OS of patients with baseline neutrophilia is poor both in NSCLC and in SCLC [11,12]. Despite limitations on reflecting the tumor microenvironment, blood-based cellular biomarkers are easier to handle than tumor tissues and have a great advantage because of specimen accessibility, quantitative measurement, the opportunity for serial monitoring, and the availability of unique analytic platforms [13].

Tumor defense can be regarded as a fine-tuned equilibrium between the destruction of cells recognized as “non-self” and the tolerance of healthy cells in the body [14]. Tolerance is maintained by multiple mechanisms, including regulatory immune cells, such as regulatory T cells or myeloid-derived suppressor cells (MDSC), immunosuppressive cytokines, and cellular ligand/receptor pairs, named immune checkpoints, which are known to down-modulate immune effector functions. By competing for the ligands or by controlling the surface expression of inhibitory immune checkpoint molecules, ICI therapy can shift the immune balance toward tumor destruction. Putative biomarkers for immunotherapy could be the number and/or the products of tolerance-inducing regulatory immune cells, which should be down-regulated with an ongoing anti-tumor immune response. On the other hand, the cells and cellular receptors involved in priming, trafficking, and target recognition of tumor-specific T cells might represent opportunities for biomarkers [14], including the number of antigen-presenting cells, such as dendritic cells (DC).

In the present study, blood immune cells were analyzed in SCLC patients undergoing combined chemo/immunotherapy. Since a high neutrophil/lymphocyte ratio (NLR), a high amount of HLA-DR^low^ MDSC, and low frequencies of 6-Sulfo LacNAc (slan)+ non-classical monocytes and DC have correlated with poor patient survival in a recent study with NSCLC patients undergoing ICI/chemotherapy [15]; we mainly focused on the analysis of these four immune cell markers. Furthermore, we compared blood immune cell parameters in SCLC and NSCLC patients to uncover the peculiarities of SCLC as a very aggressive variant of lung cancer. 

## 2. Materials and Methods

### 2.1. Patient Characteristics and General Outcome

The study was approved by the institutional review board of the Aerztekammer Sachsen-Anhalt (69/18). EDTA peripheral blood samples were obtained from patients with advanced lung cancer of SCLC histology. From February 2020 to September 2021, 40 patients with histologically confirmed locally advanced or metastatic lung cancer prior to ICI treatment with an anti-PD-L1 antibody in combination with chemotherapy were prospectively enrolled. Patients met the following criteria: age > 18 years, histologically confirmed diagnosis of advanced lung cancer, adequate organ function, and the capacity to make an informed decision. Patients with a previous history of active autoimmune disease were excluded. All patients gave written informed consent for the study proposal and procedures. The cut-off date of the study was February 2022. Patients received combined chemo/immunotherapy with carboplatin, etoposid, and atezolizumab, according to the IMpower133 trial [16]. The primary endpoint of the study was the OS of patients. The minimum follow-up for the OS (from the inclusion of the last patient to the patient’s last visit date) was 9 months. Patient’s responses were determined according to the Response Evaluation Criteria in Solid Tumors (RECIST 1.1). Patients underwent CT scans at the baseline and after 10 weeks. Subsequent assessments of the disease extent by CT scan were scheduled every 12 weeks or earlier if clinically indicated. In the case of progressive disease, patients were allowed to continue the treatment if clinical improvement was maintained, and the CT was repeated after 8 weeks to confirm progression. In addition to a RECIST-defined objective response, we assembled complete and partial clinical responses with the stable disease to obtain the disease control benefit group, which was compared to the patient’s group without durable clinical benefits. Progression-free survival (PFS) was the time elapsed from the initiation of chemo/immunotherapy until the first observation of progressive disease or death from any cause. OS was defined as the time from the initiation of chemo/immunotherapy until death from any cause. Patients who did not die or progress and those lost to follow-up were censored.

### 2.2. Blood Samples, Flow Cytometry, and Antibody Staining

Peripheral blood samples (2.6 mL EDTA monovette) were collected before the initiation of ICI/chemotherapy (time point 1, baseline) and prior to the third cycle of ICI therapy (time point 2). Leukocyte count and a complete blood count were determined using a CELL-Dyn Ruby (Abbott Lab., Wiesbaden, Germany). DC subpopulations were identified with the “Blood DC Enumeration Kit” (Cat. No. 130-091-086; Miltenyi, Bergisch Gladbach, Germany) supplemented for gating reasons with CD45-APC-H7 and an HLA-DR-V500 monoclonal antibody (mAb). Briefly, 300 µL of whole blood was incubated with a cocktail of mAbs including anti-CD1c-PE as a marker for myeloid DC (MDC), CD141/BDCA-3-APC (myeloid cDC1), and CD303/BDCA-2-FITC for plasmacytoid DC (PDC) [17]. The test kit contained an anti-CD14-mAb and CD19-PE-Cy5 to exclude monocytes and B cells from the analysis, as well as a dead-cell discriminator. After mAb incubation, erythrocyte lysis, and two washing steps, blood cells were fixed according to the manufacturer’s instructions. At least 1 million CD45+ blood leukocytes were analyzed using a recently published gating strategy [15]. Monocytic HLA-DR expression was quantified using a mAb labeled on a protein/fluorophore ratio of 1/1 (340827; clone L243; QuantiBRITE™ reagent; BD Biosciences, Heidelberg, Germany). A total of 50µL of blood was stained according to the manufacturer’s instructions. Using multi-level calibrated QuantiBRITE beads (340495; BD Biosciences, Heidelberg, Germany), a standard curve for antigen quantification was established. The measured geometric mean fluorescence intensity (MFI) of the gated population was converted into “antibody molecules bound per cell” (ABC) using a Microsoft Excel™ spreadsheet. HLA-DR MFI values of ≤5000 ABC were denoted as an “immunoparalysis” parameter in former studies that monitored immunodepression [18]. Taking an MFI of 5000 ABC as a borderline value for a low HLA-DR intensity, the number of HLA-DR^low^ monocytes was determined as a percentage of monocytes. A lysed whole blood technique with 8-color staining of blood cells was used for the labeling of lymphocytes and monocytes. A total of 300 µL of EDTA-blood was subjected to staining with mAbs specific to slan (M-DC8)-FITC (130-117-371; Miltenyi Biotec, Bergisch Gladbach, Germany); CD56-PE from Beckmann Coulter (A07788; Hamburg, Germany); CD16-PE-Cy7 from Biolegend (302016; San Diego, CA, USA); and CD19-PerCP-Cy5.5 from InVitrogen (45-0199-42; Thermo Fisher, Waltham, MA, USA). All other mAbs used, including [CD14-APC (345787), CD45-APC-H7 (560178), CD3-V450 (560365), and HLA-DR-V500 (561224)], were from BD Biosciences. The blood–antibodies mixture was incubated at room temperature for 15 min before the addition of an erythrocytes-lysing solution (349202; BD Biosciences). After 10 min of incubation and two washing steps, the cells were analyzed by flow cytometry. The gating strategy for slan+ non-classical monocytes was provided by Möller et al. [15]. Blood cell samples were measured on a FACS CANTO II Flow Cytometer (BD Biosciences, Heidelberg, Germany). Data analysis was performed using the BD FacsDIVA^TM^ software. Cytometer Setup & Tracking (CST) Beads (656047; BD Biosciences) were used daily to set the standardized geometric mean fluorescence intensity (MFI) ranges in the fluorescence channels used. 

### 2.3. Statistical Analysis

The statistical analyses were performed with the commercial software SPSS 28.0 (SPSS Inc., Munich, Germany). The median with interquartile range (IQR) was given for most data. Differences in the immune cell parameters between patient groups or between different time points were analyzed using non-parametric tests for the unpaired or paired samples, as appropriate. Accordingly, the comparison between different patient groups was based on the Mann–Whitney U test or the Chi-Square test. Survival analysis comprised a descriptive presentation of the cumulative survival functions according to Kaplan–Meier, and differences among the curves were evaluated using the log-rank test. Univariable and multivariable analyses were performed using the Cox proportional hazards model. Correlations among quantitative variables were based on the non-parametric Spearman rank correlation coefficient. For the primary outcome, a *p*-value of less than 0.05 was considered statistically significant, and the *p*-values of secondary outcomes were interpreted as exploratory.

## 3. Results

### 3.1. Patient Characteristics and General Outcome

The general baseline characteristics of the 40 SCLC patients of this study and 84 NSCLC patients of the control group are summarized in Table 1. The median age of the SCLC patients was 65 years (range, 50–87 years); most patients were male (58%) and smokers (98%). Patients underwent a mean of eight cycles of atezolizumab therapy (range 1–26). As shown in Table 1, most of the patients responded to therapy, though often only for a few months. The rate of the confirmed objective response was 85%. With a median follow-up of 21 months [95% CI 13.8–28.2], the median PFS versus OS for all the patients was 6 months [95% CI, 4.4–7.6] versus 10 months [95% CI, 8.8–11.2], respectively. Six patients (15%) stopped treatment before the third antibody application, in most cases due to clinical worsening. Patients without disease control had a mean OS of 3.2 ± 2.0 months. At the censoring date, seven patients were still on treatment.

### 3.2. Blood Cells and Therapy Response

In order to determine blood biomarkers, which predict the patient’s response to therapy, baseline blood immune cells were investigated in the patient group “progressive disease/therapy discontinuation” and the group “clinical response to therapy” (Table 2). Patients without a clinical response to therapy had a higher NLR, higher amounts of HLA-DR^low^ MDSC, a lower frequency of DC, both of MDC and PDC, as well as a lower NK cell count. No differences were found for slan+ non-classical monocytes. Predictor variables that had a significant difference between the patients’ groups with or without progress were analyzed with ROC (receiver operating characteristics) curves to determine the overall strength of association (area under the ROC curve [AUC]) and the optimal cut-off point for the prediction of therapy resistance (maximizing the sum of sensitivity and specificity). The AUC values of the ROC curves for the NLR, HLA-DR^low^ monocytes, and DC subtypes were between 0.79 and 0.89 (Table 3). The cut-off values of the risk factors were >6.1 for NLR, >21% of monocytes for HLA-DR^low^ MDSC, and < 0.05% of the leukocytes for the CD1c+ MDC. With 750 monocytes /µL blood in the mean, 21% HLA-DR^low^ monocytes correspond to 158 cells/µL blood. With 9200 leukocytes/µL blood, 0.05% DC correspond to 5 cells/µL blood. Baseline counts of leukocytes, eosinophils, erythrocytes, and platelets had no association with the patient’s response to immune/chemotherapy.

### 3.3. Comparison of Baseline and Third-Cycle Blood Cell Markers

Most of the patients with disease progression did not obtain a second blood sampling. Therefore, only the 33 patients with clinical response and with a second blood collection were chosen for the comparison of baseline and third-cycle parameters (Supplementary Table S1). An increase in neutrophils from 7600 (IQR 3500) to 9090 (6825) cells/µL (*p* = 0.043) and of lymphocytes from 1690 (1110) to 2070 (1255) cells/µL blood (*p* = 0.051) resulted in constant NLR values. The number of monocytes increased (*p* = 0.032). Other significant differences observed between the basal and third-cycle values were an increase in CD3 + T cells from 1098 (1156) to 1560 (985) cells/µL (*p* = 0.019) and an elevation of CD1c+ MDC from 0.070 (0.068) to 0.109 (0.155) percent of leukocytes (*p* = 0.004)

### 3.4. Survival Analyses

**Figure 1:**
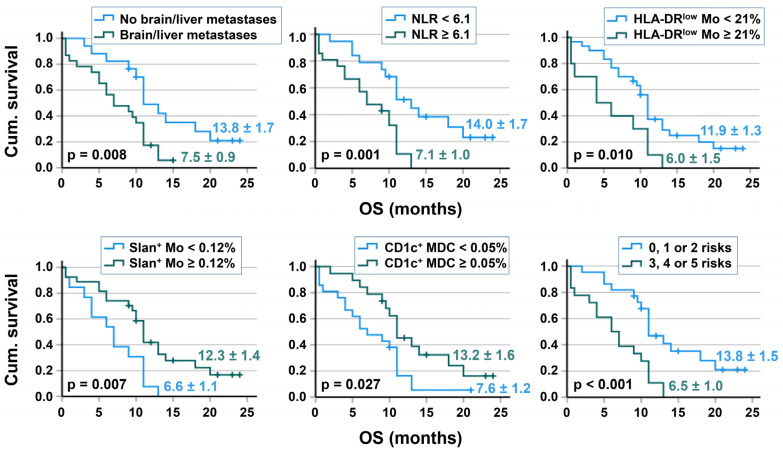
Relationship between risk factors/baseline immune cell parameters and patient’s OS. Kaplan–Meier curves are shown for the presence of brain/liver metastases, the NLR, HLA-DR^low^ MDSC (% of monocytes), slan+ non-classical monocytes (% of leukocytes), CD1c+ MDC (% of leukocytes), and a “Risk Score” of the five risks “presence of brain/liver metastases, high NLR, high amount of HLA-DR^low^ MDSC, low frequency of slan+ non-classical monocytes, and low CD1c+ MDC”. Mean survival time and *p*-value of the log-rank test are given.

The global median OS of patients was 10.4 ± 1.1 months (95% confidence interval (CI): 8.85–11.15), with ten patients (25%) demonstrating an OS of at least 12 months. Kaplan–Meier analyses were performed to analyze survival differences based on several risk factors, including the presence of brain/liver metastases and the baseline immune cell repertoire. Compared to the respective reference group, a better OS was found for 17 patients without any brain/liver metastases, 19 patients with an NLR < 6.1, 30 patients with a frequency of HLA-DR^low^ MDSC < 21% of monocytes, 27 patients with ≥ 0.12% slan+ non-classical monocytes (as % of leukocytes), and 21 patients with CD1c+ MDC ≥ 0.05% of leukocytes. The hazard ratio for OS was 3.04 (1.45–6.99) for the NLR, 2.48 (1.15–5.33) for HLA-DR^low^ MDSC, 2.51 (1.20–5.24) for slan+ non-classical monocytes and 2.08 (1.03–4.2) for MDC (Table 4). Patients with zero–two risk factors had a significantly better OS compared to patients with three–five risk factors. No relevant survival differences were found for basal PDC frequencies, as well as for the numbers of T-, B-, and NK cells. The results of univariable prognostic factor analysis (Kaplan–Meier and Cox regression) are provided in Table 4. Kaplan–Meier pictures are shown in Figure 1. 

**Figure 2:**
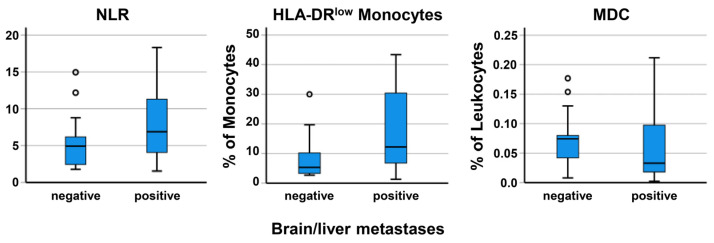
Comparison of baseline immune parameters in SCLC patients with or without brain/liver metastases. The observed differences are significant in the case of HLA-DR^low^ monocytes (*p* = 0.034). The whiskers of box plots indicate the largest/lowest points inside the range defined by first or third quartile plus 1.5 times interquartile range (IQR). The circles represent outliers.

In a multivariable Cox regression analysis of OS, considering the covariate status of brain/liver metastases, only the NLR baseline values were an independent prognostic factor (*p* = 0.042). Comparing blood parameters in the 17 patients without versus the 23 patients with brain/liver metastases, no significant differences were detected for the number of neutrophils, lymphocytes, slan+ non-classical monocytes, and the DC subtypes. The metastasis group had a higher frequency of HLA-DR^low^ MDSC (12.2% versus 5.3%, *p* = 0.034) and a tendency to both a higher NLR (6.9 versus 4.9, *p* = 0.066) and lower MDC (0.03% versus 0.07% of leukocytes), as illustrated in Figure 2. Data are provided in Supplementary Table S2.

To obtain a better insight into whether the baseline immune cell parameters—irrespective of primary therapy resistance—correlate with survival, we repeated survival analyses in the 34 patients who responded to therapy (Supplementary Table S3). Again, the patients with brain/liver metastases or a baseline NLR ≥ 6.1 had a significantly worse OS with <0.12% slan+ non-classical monocytes. No significant differences were found for HLA-DR^low^ MDSC and the different DC subtypes. Our results suggest that the basal abundance of HLA-DR^low^ MDSC might be involved in primary therapy resistance to chemo/immunotherapy in SCLC patients. By contrast, slan+ non-classical monocytes could represent a factor that is important for long-lasting therapy response and survival. Additional factors, such as the presence of brain/liver metastases, also had an impact. The 11 SCLC patients without any brain/liver metastases and with an NLR < 6.1 had a mean OS of 16.9 (13.4–20.3) months. Already one of both risk factors resulted in a significantly shorter OS (8.2 months) (*p* < 0.001).

To investigate whether immune cell parameters at the third cycle of therapy better correlated with the patient’s OS, we repeated survival analyses with the blood parameters of the third cycle (n = 35). As shown in Supplementary Table S4, neutrophil counts and the NLR lost their impact on OS, whereas DC subtypes (PDC and MDC) became more strongly correlated with OS. 

### 3.5. Correlation of Immune Cell Subpopulations

The baseline neutrophil counts directly correlated with the monocyte counts. Neutrophil numbers correlated even more strongly with the percentages of HLA-DR^low^ MDSC (Table 5). Neutrophil numbers did not correlate with lymphocyte counts. The neutrophil counts indirectly correlated with the percentages of slan+ non-classical monocytes and with DC, whereby the correlation with CD1c+ MDC was stronger than the correlation with PDC. Correlations between the immune cell subpopulations were also found during the third therapy cycle and were often even stronger (Table 5).

### 3.6. Comparison of Immune Cell Parameters in Patients with SCLC and NSCLC

**Figure 3:**
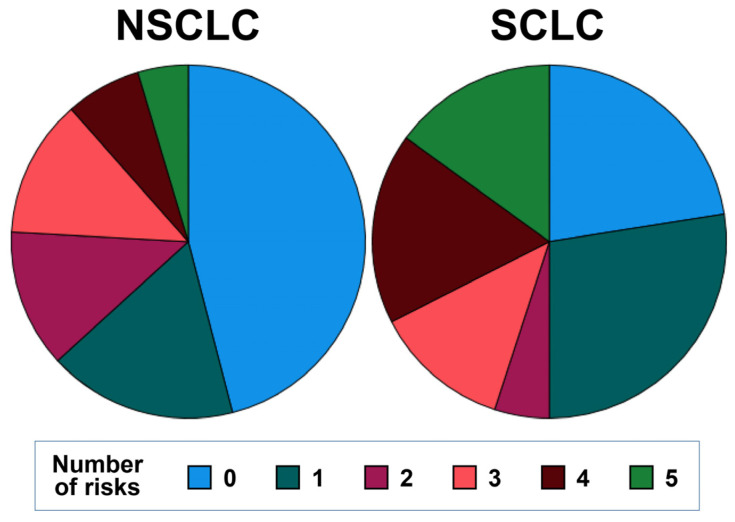
Comparison of the basal number of risk factors (presence of brain/liver metastases, high NLR, high amount of HLA-DR^low^ MDSC, low frequency of slan+ non-classical monocytes, and low CD1c+ MDC) between NSCLC and SCLC.

Despite the patient’s ages being comparable in SCLC and NSCLC (Table 1), SCLC patients had a significantly higher frequency of brain/liver metastases (55% in SCLC, 26% in NSCLC). Comparing the baseline blood immune cell parameters, a significantly lower amount of MDC and PDC was detected in SCLC (Supplementary Table S5). Furthermore, SCLC patients tended to have a higher NLR. No significant differences could be found for HLA-DR^low^ MDSC, slan+ non-classical monocytes, and T-, B-, and NK cells. The number of risk factors differed significantly between the patients of the two histotypes. Figure 3 illustrates that 46% of NSCLC patients, compared to 22.5% of SCLC had no basal risk factor with respect to the five risk factors “brain/liver metastasis, high NLR, high amount of HLA-DR^low^ MDSC, low frequency of slan+ non-classical monocytes and of CD1c+ MDC (cut-off values of SCLC). Otherwise, 45% of SCLC patients, compared to only 24% of NSCLC patients, had three–five risk factors. Both in SCLC and NSCLC, the neutrophil numbers were directly correlated with monocyte counts, especially with the frequency of HLA-DR^low^ MDSC. Furthermore, neutrophils and the NLR were indirectly correlated with slan+ non-classical monocytes and with DC subpopulations in both histotypes.

With respect to therapy response, higher neutrophil counts were associated with a lack of therapy response and progressive disease in the case of NSCLC patients [15]. The baseline neutrophil counts of SCLC patients did not differ significantly between therapy responders and non-responders, but the NLR of SCLC patients was significantly higher in the non-responder group (Table 2). HLA-DR^low^ MDSC did not differ between therapy responders and non-responders in the case of NSCLC patients [15] but were significantly higher in SCLC patients with progress. Those patients also had the lowest NK cell numbers. A significantly lower MDC/PDC sum in patients with progress could be observed both for patients with SCLC and NSCLC.

To compare the survival data of SCLC and NSCLC patients, the risk factors of SCLC patients were applied to the OS of NSCLC patients. Brain/liver metastasis had a weak effect on the OS of NSCLC patients, and Kaplan–Meier curves showed separate lines only after 14 months, as illustrated in Supplementary Figure S1. Both in SCLC and NSCLC patients, the NLR and the frequency of slan+ non-classical monocytes, and CD1c+ MDC significantly correlated with OS. Patients with <21% HLA-DR^low^ MDSC showed a tendency for better OS (*p* = 0.055) in NSCLC. A total of 61 NSCLC patients with zero–two risks (of the five risks: brain/liver metastases, high NLR, high level of HLA-DR^low^ MDSC, low amounts of slan+ non-classical monocytes and low CD1c+ MDC) had a significantly better OS than 19 patients with three–five risks (Supplementary Figure S1).

## 4. Discussion

SCLC is a lung cancer subtype with a particularly poor prognosis because of a strong predilection for early metastasis and therapeutic resistance. SCLC has one of the highest rates of mutational burden, suggesting that this cancer type is particularly susceptible to immune-based therapeutic approaches [19]. Prior findings that have associated a higher number of tumor-infiltrating immune cells with improved SCLC outcomes support this view [20,21]. Platinum-based chemotherapy with or without ICI is currently a first-line therapy for SCLC patients, despite the heterogeneous outcome [2,3,4,5,9]. The application of ICI therapy in SCLC appears to be less effective when compared to NSCLC, and only a minority of SCLC patients benefit [22]. Efforts to obtain a more comprehensive knowledge of how different cell types interact with each other during ICI therapy and ultimately affect clinical response is still an ongoing task. The greatest obstacles to the optimal success of immunotherapy remain a large percentage of partial responders (primary resistance) and the high rate of resistance acquisition. The mechanisms of immunotherapy resistance remain poorly understood. Both tumor cell-intrinsic (lack of tumor antigens, disturbed antigen presentation, genetic T cell exclusion) and tumor cell-extrinsic (absence of T cells, inhibitory immune checkpoints, immunosuppressive cells) factors contribute to immunotherapy resistance (for review, see [23]). A combination of agents with different mechanisms of action is one major strategy to overcome resistance mechanisms and to maximize the benefits of immunotherapy (for review, see [24]). 

On the other hand, biomarkers have to be developed to select potential responders or to exclude potential non-responders. In NSCLC, several papers exploring predictive biomarkers for a response to ICI have been published [7,14,25,26,27,28]. NSCLC patients responding to ICI/chemotherapy have already at the baseline a favorable immune profile with a low baseline NLR, a low number of HLA-DR^low^ MDSC, and higher levels of slan+ non-classical monocytes and DC, correlating with longer survival [15]. A similar picture could be observed in the SCLC patients of the current study, though with some peculiarities. A high NLR was strongly associated both with primary resistance to therapy and with poor OS. The presence of brain/liver metastases correlated with poor OS in SCLC but scarcely in NSCLC patients. All SCLC patients without a therapy response had brain/liver metastases associated with a higher frequency of HLA-DR^low^ MDSC. HLA-DR^low^ MDSC seemed to be associated more with primary than with a late-acquired therapy resistance since the impact of these blood parameters on the OS of a subgroup of patients responding to therapy was limited. A lower NLR was associated with a higher frequency of slan+ non-classical monocytes and correlated with better OS. In patients responding to therapy, the combined chemo/immunotherapy resulted in an increase in CD3+ T cells at the time point of the third therapy cycle. Nevertheless, no correlation was found between the number of lymphocytic subpopulations and survival. Compared to NSCLC, SCLC patients had a more suppressed state of blood DC (both of MDC and PDC) and more risk factors at the baseline, such as the presence of brain/liver metastases, high NLR, a high amount of HLA-DR^low^ MDSC, low amounts of slan+ non-classical monocytes, and low amounts of CD1c+ MDC. While the frequency of basal PDC correlated with survival in NSCLC, only the third cycle and not the baseline frequencies of PDC did this in SCLC.

The OS of SCLC patients with baseline neutrophilia has been shown to be poor [11,29]. During chronic inflammatory processes, such as malignancy, there is a persistent signal to recruit neutrophils and monocytes from the bone marrow. Tumor cells produce the granulocyte colony-stimulating factor (G-CSF), which skews the neutrophil retention/release balance in the bone marrow, leading to increased neutrophils in the blood [30]. Suppressive neutrophils, which are the granulocytic arm of MDSC, promote tumor progression by contributing to genetic instability, tumor cell proliferation, and angiogenesis (for review, see [31]). Furthermore, neutrophils can dampen anti-tumor immunity by suppressing T-cell proliferation, cytokine secretion, and the cytotoxic activity of activated T cells and natural killer cells [32]. Targeting MDSC via all-trans-retinoic acid can improve the induction of immune responses by a cancer vaccine in SCLC [33]. Neutrophils might represent an escape mechanism that is linked to the resistance against ICI therapy and to poor patient outcomes (for review, see [34]). Blood neutrophils and the derived NLR are established biomarkers of therapy response. A pretreatment NLR < 5 was associated with longer OS in patients who had several advanced cancers undergoing therapy with different ICI variants [35]. Our result of a correlation of a high NLR with a poor prognosis in SCLC patients corroborates the data of other investigators (for meta-analysis, see [36]). SCLC patients even had higher NLR values than those observed for NSCLC. Rice and Belani compared blood-based biomarkers and indicators for systemic inflammation in NSCLC and SCLC patients, but these authors described higher levels of systemic inflammation in NSCLC, including a higher NLR [37]. The cause of this discrepancy with our data remains unclear. 

In the current study, neutrophil numbers positively correlated with monocyte counts and with the percentage of HLA-DR^low^ MDSC, as earlier described for NSCLC patients of different tumor stages [38]. Circulating monocytes are the precursors of essential myeloid cells, such as tumor-associated macrophages, MDSC, and DC. HLA-DR^low^ monocytes are known to suppress the functions of lymphocytes in cancer patients [39,40], similar to the situation described in sepsis [41] and poly-trauma [42]. HLA-DR is one of three MHC class II glycoproteins expressed on antigen-presenting cells whose function is to present tumor peptide antigens to the T-cell receptors on CD4+ T cells resulting in cellular activation. As such, HLA-DR^low^ monocytes have a diminished capacity to present antigens to T cells. Furthermore, MDSC suppress immune cells by expressing PD-L1 [43], by secreting IL-10 and transforming growth factor (TGF)β [44,45], or by showing a deficient generation of mature DC [46]. Monocytic MDSC is known to impede the treatment response to ICI and, compared to polymorphonuclear MDSC, might even have a stronger prognostic value in NSCLC patients [47]. In the current study, primary therapy resistance was associated with the highest frequencies of HLA-DR^low^ MDSC, and especially patients with brain/liver metastases expressed huge amounts of this MDSC type. While an age-matched control group has 2.6 ± 2.5% of HLA-DR^low^ monocytes [38], tenfold higher amounts could be found in patients with progressive SCLC. The high levels of HLA-DR^low^ MDSC might suggest that those cancer patients have reached a point of immunoparalysis prior to treatment and thus may not be responsive to immunotherapeutic approaches. Interestingly, platinum agents as the backbone of chemotherapy for metastatic lung cancer can not only increase antigen presentation by cancer cells and promote T cell trafficking into the tumor microenvironment but could also diminish HLA-DR^low^ MDSC [48,49]. Comparing baseline and third cycle values of HLA-DR^low^ monocytes, we could not observe a decline in these MDSC in SCLC patients undergoing therapy, though the further time course has not been considered. The detailed mechanisms of monocytic reprogramming by cancer therapy still have to be elucidated [50]. 

In the current study, HLA-DR^low^ MDSC and slan+ non-classical monocytes were investigated as two monocytic subpopulations with contrasting properties. Both types of monocytes even showed an inverse correlation in SCLC, similar to that earlier described for NSCLC [15]. CD16+ non-classical monocytes can be further divided into slan+ and slan-negative populations [51,52]. While being of monocyte origin, slan+ cells either rapidly acquire DC functions or differentiate into macrophages [53]. Slan+ non-classical monocytes have been shown to be involved in anti-tumoral activity [53] since they can activate NK cells via IL-12. The crosstalk between slan+ cells and NK cells improves the differentiation of naïve CD4+ T lymphocytes into interferon (IFN)-gamma-producing TH1 cells [54]. The median number of slan+ cells in SCLC patients fits the amount of slan+ cells reported in the literature for a control group (21 cells/µL, [55]). The frequency of baseline slan+ monocytes was not useful as a marker of treatment failure but correlated with the survival both of SCLC and NSCLC patients in the current study. 

Although the frequency of slan+ non-classical monocytes directly correlated with DC subtypes in both SCLC (current study) and NSCLC [15], several differences were noticed between DC and slan+ non-classical monocytes. From the baseline to the third cycle, the frequency of all blood DC subpopulations rose in patients with a clinical response, but slan+ non-classical monocytes stayed stable over time. SCLC compared to NSCLC patients, had similar levels of slan+ non-classical monocytes (median 0.23% of leukocytes) but significantly lower DC concentrations. The MDC/PDC median was 0.10% of leukocytes in SCLC; however, it was 0.20% in advanced NSCLC. The observation of significantly lower blood DC in SCLC confirms the data published by Afifi and coauthor [56]. In SCLC therapy responders, basal slan+ non-classical monocytes but not DC levels correlated with patients’ OS. In particular, basal PDC frequencies were not associated with OS in our study. However, in the third cycle of ICI administration, all DC subpopulations were significantly correlated with survival. The impact of these observations remains unclear. Some of the SCLC patients had very low basal amounts of blood DC, which might contribute to their disturbed immune functions and poor prognosis, suggesting that a critical minimum number of DC is not reached in the case of basal PDC. In a recent study, an age-matched control group had 7.5 ± 4.2 PDC/µL blood [38]; in the current study, the baseline PDC of SCLC patients was 0.3–5.0 in the progression group, and 0–17.2 PDC/µL of blood in the clinical response, with a median of 4.9 PDC/µL blood. Although DC constitutes a rare immune cell population, these cells are central for the initiation of tumor antigen-specific immunity [57]. Systemic effects induced by lung cancer cells upon circulating DC may result in diminished and functionally handicapped cells [58]. DC in NSCLC tissues upregulates the co-inhibitory receptor B7-H3/CD276, thus failing to stimulate T lymphocytes [59]. DC dictates responses to ICI treatment since a DC gene signature was strongly associated with the improved OS of NSCLC patients [60]. Until now, data on DC in SCLC patients have been scarce. Understanding the possibilities to augment DC functions could offer new approaches to enhance the efficacy of immunotherapy. 

In addition to the combination of ICI with other therapies, several new treatment strategies for SCLC are under investigation with promising results [61]. Despite notable clinical responses, basic and clinical studies are still required to investigate the exact mechanism of response to ICI therapy and to improve the appropriate selection of patients. Identifying low percentages of both slan+ non-classical monocytes and CD1c+ MDC as well as a high NLR and high percentages of HLA-DR^low^ MDSC as risk factors for the patient’s response to a combined chemo/immunotherapy, this study extends our knowledge of biomarkers and the pathophysiological causes of therapy resistance. If validated in larger studies, biomarker analysis in blood samples could help to select SCLC patients for a higher benefit from immunotherapy. 

## Figures and Tables

**Table 1:** Patient characteristics and clinical response to therapy.

	SCLC	NSCLC (AC)	NSCLC (SqC)
Number	40	57	27
Age, median (IQR)	65 (9)	64 (15)	67 (8)
Sex			
Male, n (%)	23 (57.5)	36 (63.2)	25 (92.6)
Female, n (%)	17 (42.5)	21 (36.8)	2 (7.4)
ECOG, n (%)			
0	10 (25)	35 (61.4)	14 (51.85)
1	26 (65)	22 (38.6)	13 (48.15)
2	4 (10)	0	0
Smoker status			
-Never-smoker	1 (2.5)	11 (19.3)	1 (3.7)
-Smoker	39 (97.5)	46 (80.7)	26 (96.3)
Metastases, n (%)			
<3	17 (42.5)	26 (45.6)	17 (63)
≥3	23 (57.5)	31 (54.4)	10 (37)
Brain and/or liver metastases n (%)	22 (55)	16 (28.6)	6 (22.2)
Therapy setting: Chemotherapy	Carboplatin + Etoposid	Carboplatin + pemetrexed (TTF-1+) or + nab-Paclitaxel (TTF-1neg.)	Carboplatin + nab-Paclitaxel
Therapy setting: ICI + others	Atezolizumab	Pembrolizumab or (if liver metastasis) Atezolizumab + Bevacizumab	Pembrolizumab
Radiation before ICI, n (%)	4 (10)	6 (10.5)	3 (11.1)
Radiation after ICI, n (%)	15 (37.5)	9 (15.8)	7 (25.9)
Clinical response, n (%)			
-Progression/Discontinuation	6 (15)	14 (25)	8 (29.6)
-Disease stabilization	3 (7.5)	10 (18)	2 (7.4)
-Partial/complete response	31 (77.5)	32 (59)	17 (63)

AC—adenocarcinoma; SqC—squamous cell carcinoma; ICI—immune checkpoint inhibitor; IQR—interquartile range; TTF—thyroid transcription factor.

**Table 2:** Baseline blood immune cell parameters. Patients with SCLC were grouped into progress/therapy discontinuation and clinical response (stabilization of disease, or partial/complete remission). Median and IQR are given as well as significant differences of Mann-Whitney U-test.

Parameters	Progressive Disease/ Therapy Discontinuation	Clinical Response	*p* Value
n	6	34	
Neutrophil counts (cells/μL)	12,950 (9530)	7540 (3560)	
Lymphocyte counts (cells/μL)	983 (1168)	1625 (1073)	
NLR	9.3 (6.5)	5.0 (6.5)	0.024
CD3+ T cells	672 (854)	1075 (1108)	
CD19+ B cells	194 (176)	178 (181)	
NK cells	67.5 (95)	237.5 (237)	0.010
Monocytes (cells/μL)	748 (478)	840 (340)	
HLA-DR^low^ MDSC (% of monocytes)	30.5 (16.1)	7.9 (22.1)	0.008
Slan+ non-classical monocytes (% of leukocytes)	0.17 (0.30)	0.16 (0.32)	
CD1c+ MDC (% of leukocytes)	0.013 (0.033)	0.062 (0.074)	0.019
CD141+ MDC (% of leukocytes)	0.001 (0.001)	0.004 (0.005)	0.001
CD303+ PDC (% of leukocytes)	0.0095 (0.019)	0.067 (0.068)	0.021

**Table 3:** Receiver operating characteristic curve analysis for the prediction of therapy failure/progress by baseline immune cell parameters.

Prediction Variable at Baseline	Cutoff Point	AUC	95% CI	*p* Value
NLR	6.1	0.789	0.645–0.934	0.025
NK cells (cells/µL)	150	0.824	0.671–0.976	0.012
HLA-DR^low^ MDSC (% of monocytes)	21	0.831	0.649–1.000	0.011
Slan+ non-classical monocytes (% of leukocytes)	0.12	0.576		
CD1c+ MDC (% of leukocytes)	0.05	0.799	0.644–0.954	0.021
CD141+ MDC (% of leukocytes)	0.0015	0.887	0.782–0.992	0.003
CD303+ PDC (% of leukocytes)	0.014	0.792	0.628–0.956	0.024

AUC—area under the ROC curve; CI—confidence interval.

**Table 4:** Relationship between baseline blood immune cell parameters with patient’s survival for 40 SCLC patients (A PFS; B OS). Data of univariate prognostic factor analysis are provided, with estimated mean of survival ± standard error, hazard ratios (HR) with 95% confidence interval (CI), and *p*-values.

A	Cut-Off	n	Kaplan–Meier PFS			Cox Regression, PFS		
			% Censored	PFS (Months)	*p* Value	HR	95% CI	*p* Value
Neutrophil counts (cells/μL)	≤10,000	30	23.3	10.2 ± 1.4	0.002		1.44–6.88	0.004
	>10,000	10	0	4.0 ± 1.1		3.14		
NLR	<6.1	19	31.6	12.3 ± 1.8	0.001		1.46–6.54	0.003
	≥6.1	21	4.8	5.1 ± 0.77		3.04		
HLA-DR^low^ MDSC (% of monocytes)	<21	30	23.3	10.0 ±1.4	0.012		1.14–5.19	0.021
	≥21	10	0	4.4 ± 1.3		2.44		
Slan+ monocytes (% of leukocytes)	<0.12	13	0	5.6 ± 0.93	0.038	2.03	0.99–4.16	0.053
	≥0.12	27	25.9	10.2 ± 1.6				
CD1c+ MDC (% of leukocytes)	<0.05	19	9.5	5.4 ± 0.8	0.017	2.24	1.09–4.57	0.028
	≥0.05	21	26.3	11.4 ± 1.7				
Baseline risk score	0–2 risk factors	22	31.8	12.1 ± 1.7	<0.001		1.67–7.06	<0.001
	3–5 risk factors	18	0	4.6 ± 0.8		3.43		
**B**	**Cut-off**	**n**	**Kaplan–Meier OS**			**Cox Regression, OS**		
			**% Censored**	**OS (Months)**	***p*** **Value**	**HR**	**95% CI**	***p*** **Value**
Neutrophil counts (cells/μL)	≤10,000	30	23.3	12.2 ± 1.2	<0.001		1.75–8.89	<0.001
	>10,000	10	0	5.2 ± 1.3		3.95		
NLR	<6.1	19	31.6	14.0 ± 1.7	0.001		1.45–6.99	0.004
	≥6.1	21	4.8	7.1 ± 1.0		3.18		
HLA-DR^low^ MDSC (% of monocytes)	<21	30	23.3	11.9 ± 1.3	0.010		1.15–5.33	0.020
	≥21	10	0.0	6.0 ± 1.5		2.48		
Slan+ monocytes (% of leukocytes)	<0.12	13	0	6.7 ± 1.1	0.007	2.51	1.20–5.24	0.014
	≥0.12	27	25.9	12.3 ± 1.4				
CD1c+ MDC (% of leukocytes)	<0.05	19	9.5	7.6 ± 1.2	0.027	2.08	1.03–4.20	0.041
	≥0.05	21	26.3	13.2 ± 1.6				
Baseline risk score	0–2 risk factors	22	31.8	13.8 ± 1.5	<0.001		1.54–6.76	0.002
	3–5 risk factors	18	0	6.5 ± 1.04		3.23		

**Table 5:** Association of blood immune cell parameters analyzed by Spearman’s rank correlation.

Baseline Blood Immune Cells	Correlation Coefficient	*p* Value
Neutrophil number with monocyte count	0.560	<0.001
Neutrophil number with percentage of HLA-DR^low^ MDSC	0.571	<0.001
Neutrophil number with frequency of slan+ non-classical monocytes	−0.629	<0.001
Neutrophil number with frequency of CD1c+ MDC	−0.610	<0.001
Neutrophil number with frequency of CD303+ PDC	−0.463	0.003
HLA-DR^low^ MDSC with frequency of slan+ non-classical monocytes	−0.527	<0.001
HLA-DR^low^ MDSC with frequency of CD1c+ MDC	−0.655	<0.001
HLA-DR^low^ MDSC with frequency of CD303+ PDC	−0.629	<0.001
slan+ non-classical monocytes with frequency of CD1c+ MDC	0.506	<0.001
**Blood immune cells at third cycle of therapy**		
Neutrophil number with percentage of HLA-DR^low^ MDSC	0.553	<0.001
Neutrophil number with frequency of slan+ non-classical monocytes	−0.691	<0.001
Neutrophil number with frequency of CD1c+ MDC	−0.727	<0.001
HLA-DR^low^ MDSC with frequency of slan+ non-classical monocytes	−0.767	<0.001
HLA-DR^low^ MDSC with frequency of CD1c+ MDC	−0.663	<0.001
slan+ non-classical monocytes with frequency of CD1c+ MDC	0.721	<0.001

## Data Availability

The data will be available after publication on request.

## Supplementary Materials

The following supporting information can be downloaded at: https://www.mdpi.com/article/10.3390/biom13020190/s1, **Figure S1**: Relationship between risk factors/baseline immune cell parameters and OS in 84 NSCLC patients based on cutoffs of the SCLC group. Mean survival time and *p*-value of the log-rank test are given; **Table S1**: Comparison of baseline and third cycle values of blood immune cells in 33 SCLC patients with therapy response to immune/chemotherapy. The median and interquartile ranges are given. Bold values highlight significant differences in the Wilcoxon test; **Table S2**: Comparison of blood immune cells in SCLC patients without and with brain/liver metastases. The median and interquartile ranges are given. Bold values highlight significant differences in Mann–Whitney U test; **Table S3**: Relationship between baseline immune-cell parameters with patients’ OS for 34 SCLC patients responding to chemo/immunotherapy. Data of univariate prognostic factor analysis are provided with estimated mean of survival ± standard error, hazard ratios (HR) with 95% confidence interval (CI), and *p*-values; **Table S4**: Relationship between cycle 3 blood immune cell parameters with patients’ OS for 35 SCLC patients. Data of univariate prognostic factor analysis is provided, with estimated mean of survival ± standard error, hazard ratios (HR) with 95% confidence interval (CI), and *p*-values; **Table S5**: Baseline blood immune cell parameters of SCLC compared to NSCLC histotypes. Data represent the median and interquartile range (IQR). The results of the Mann–Whitney U-test are shown.
