# Supplementary material for: Monitoring Blood Immune Cells in Patients with Advanced Small Cell Lung Cancer Undergoing a Combined Immune Checkpoint Inhibitor/Chemotherapy
Source: Biomolecules. 2023 Jan 17;13(2):190. doi: 10.3390/biom13020190 (PMC9953684; doi:10.3390/biom13020190)
Supplement: Supplementary file 1 [file biomolecules-13-00190-s001.zip › biomolecules-2113847-supplementary.pdf]

Suppl. Figure S1. Relationship between risk factors/baseline immune cell parameters and OS in 84 NSCLC patients based on cutoffs of the SCLC group. Mean survival time and p value of the log rank test are given.

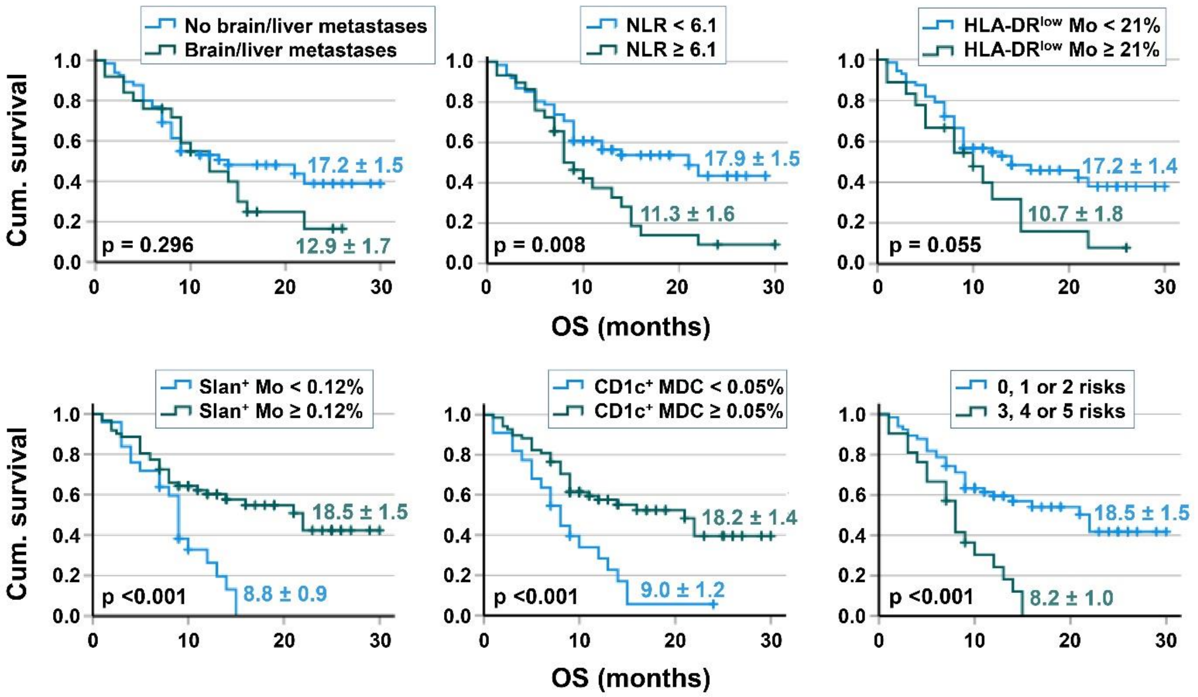

Table S1. Comparison of baseline and third cycle values of blood immune cells in 33 SCLC patients with therapy response to chemo/immunotherapy. Median and interquartile range are given. Bold values highlight significant differences in Wilcoxon test.

Parameters	Baseline	Third cycle	*P value*
Neutrophil counts (cells/µl)	7600 (3500)	9090 (6825)	**0.043**
Lymphocyte counts (cells/µl)	1690 (3500)	2070 (1255)	0.051
NLR	5.1 (6.7)	4.9 (3.8)	
CD3+ T cells	1098 (1156)	1560 (985)	**0.019**
CD19+ B cells	180 (172)	163 (190)	
NK cells	245 (242)	198 (160)	
Monocytes (cells/µl)	660 (467)	850 (340)	**0.032**
HLA-DR^{low} MDSC (% of monocytes)	7.4 (10.4)	8.5 (13.3)	
Slan+ non-classical monocytes (% of leukocytes)	0.227 (0.485)	0.219 (0.376)	
CD1c+ MDC (% of leukocytes)	0.07 (0.068)	0.109 (0.155)	**0.004**
CD141+ MDC (% of leukocytes)	0.004 (0.005)	0.006 (0.006)	
CD303+ PDC (% of leukocytes)	0.051 (0.072)	0.069 (0.074)	
Sum of MDC/PDC (% of leukocytes)	0.126 (0.147)	0.186 (0.187)	**0.008**

Table S2. Comparison of blood immune cells in SCLC patients with or without brain/liver metastases. Median and interquartile range are given. Bold values highlight significant differences in Mann Whitney U test.

Parameters	No brain/liver metastases	brain/liver metastases	*P value*
n	17	23	
Neutrophil counts (cells/µl)	6880 (2555)	9050 (8420)	
Lymphocyte counts (cells/µl)	1560 (1075)	1560 (1034)	
NLR	4.9 (4.4)	6.9 (8.9)	0.066
CD3+ T cells	1136 (1112)	1024 (922)	
CD19+ B cells	186 (176)	170 (167)	
NK cells	255 (252)	201 (179)	
Monocytes (cells/µl)	665 (501)	660 (510)	
HLA-DR^{low} MDSC (% of monocytes)	5.3 (9.1)	12.2 (24.5)	**0.034**
Slan+ non-classical monocytes (% of leukocytes)	0.30 (0.36)	0.19 (0.61)	
CD1c+ MDC (% of leukocytes)	0.07 (0.048)	0.031 (0.079)	
CD141+ MDC (% of leukocytes)	0.004 (0.003)	0.002 (0.005)	
CD303+ PDC (% of leukocytes)	0.05 (0.08)	0.05 (0.06)	
Sum of MDC/PDC (% of leukocytes)	0.124 (0.111)	0.086 (0.135)	

Table S3. Relationship between baseline immune-cell parameters with patients' OS for 34 SCLC patients responding to chemo/immunotherapy. Data of univariate prognostic factor analysis is provided with estimated mean of survival ± standard error, hazard ratios (HR) with 95% confidence interval (CI) and *p* values.

	Cutoff	n	Kaplan-Meier OS			Cox Regression, OS		
			% cen-sored	OS (months)	*p value*	HR	95% CI	*p value*
Neutrophil counts (cells/µl)	≤10,000	28	25	12.6 ± 1.28	**0.018**		1.1-7.5	**0.031**
	>10,000	6	0	7.67 ± 1.2		2.87		
NLR	<6.1	19	31.6	14.0 ± 1.69	**0.009**		1.18-6.26	**0.019**
	≥6.1	15	6.7	8.67 ± 0.77		2.72		
HLA-DR^{low} MDSC (% of monocytes)	<21	29	24.1	12.3 ± 1.26	0.112		0.77-5.66	0.150
	≥21	5	0	8.2 ± 1.39		2.084		
Slan+ monocytes (% of leukocytes)	<0.12	10	0	8.2 ± 1.05	**0.023**	2.36	1.04-5.36	**0.040**
	≥0.12	24	29.2	13.3 ± 1.44				
CD1c+ MDC (% of leukocytes)	<0.05	15	13.3	9.4 ± 1.19	0.140	1.70	0.79-3.67	0.173
	≥0.05	19	26.3	13.2 ± 1.56				
Baseline risk score (5 factors)	0-2 risk factors	22	31.8	13.76 ± 1.53	**0.006**		1.24-6.31	**0.013**
	3-5 risk factors	12	0	8.17 ± 0.9		2.80		

Table S4. Relationship between third-cycle blood immune cell parameters with patients' OS for 35 SCLC patients. Data of univariate prognostic factor analysis is provided, with estimated mean of overall survival ± standard error, hazard ratios (HR) with 95% confidence interval (CI) and *p* values.

	Cutoff	n	Kaplan-Meier OS			Cox Regression, OS		
			% censored	OS (months)	*p value*	HR	95% CI	*p value*
Neutrophil counts (cells/µl)	≤10,000	21	28.6	13.2 ± 1.4	0.076		0.88-3.93	0.101
	>10,000	14	7.1	9.2 ± 1.4		1.86		
NLR	<6.1	23	26.1	13.3 ± 1.4	**0.045**		0.94-4.61	0.07
	≥6.1	12	8.3	8.5 ± 1.15		2.08		
HLA-DR^{low} MDSC	<21	31	22.6	12.4 ± 1.1	**0.011**		1.17-10.4	**0.025**
(% of monocytes)	≥21	4	0	5.8 ±1.9		3.49		
Slan+ monocytes (% of leukocytes)	<0.12	13	7.7	8.6 ± 1.3	**0.018**	2.32	1.08-5.0	**0.032**
	≥0.12	22	27.3	13.5 ± 1.4				
CD1c+ MDC (% of leukocytes)	<0.05	9	0	6.8 ± 1.3	**<0.001**	3.51	1.53-8.04	**0.003**
	≥0.05	29	26.9	13.4 ± 1.25				
CD141+ MDC (% of leukocytes)	<0.0015	7	0	5.9 ± 1.45	**<0.001**	3.86	1.59-9.37	**0.003**
	≥0.0015	28	25	12.9 ± 1.2				
PDC (% of leukocytes)	<0.014	6	0	5.7 ± 1.45	**<0.001**	4.57	1.76-11.88	**0.002**
	≥0.014	29	24.1	12.9 ± 1.2				

Table S5. Baseline blood immune cells parameters of SCLC compared to NSCLC histotypes. Data represent median and interquartile range (IQR), results of Mann-Whitney U-test are shown.

	SCLC	NSCLC	*P value*
n	40	83	
Neutrophil counts (cells/μl)	7680 (5155)	6590 (5330)	
Lymphocyte counts (cells/μl)	1560 (1018)	1650 (740)	
NLR	6.12 (7.1)	3.96 (4.42)	**0.04**
T cells (cells/μl)	1051 (1024)	1110 (690)	
B cells (cells/μl)	178 (172)	111 (156)	0.05
NK cells (cells/μl)	204 (226)	255 (310)	**0.009**
Monocytes	660 (483)	705 (356)	
HLA-DR^{low} MDSC (% of monocytes)	9.6 (17.2)	6.9 (14.2)	
Slan+ non-classical monocytes (% of leukocytes)	0.23 (0.46)	0.23 (0.45)	
CD1c+ MDC (% of leukocytes)	0.048 (0.069)	0.099 (0.096)	**0.002**
CD141+ MDC (% of leukocytes)	0.003 (0.005)	0.006 (0.006)	**<0.001**
PDC (% of leukocytes)	0.05 (0.07)	0.077 (0.091)	**<0.001**
MDC/PDC sum	0.10 (0.13)		
